# Flunarizine related movement disorders: a nationwide population-based study

**DOI:** 10.1038/s41598-018-37901-z

**Published:** 2019-02-08

**Authors:** Kai-Ming Jhang, Jing-Yang Huang, Oswald Ndi Nfor, Yu-Chun Tung, Wen-Yuan Ku, Cheng-Feng Jan, Yung-Po Liaw

**Affiliations:** 1 Department of Public Health and Institute of Public Health, Chung Shan Medical University, Taichung City, Taiwan; 2 Department of Neurology, Lu-Tung Christian Hospital, Changhua, Taiwan; 3 Department of Pharmacy, Taichung Veterans General Hospital, Taichung, Taiwan; 4 Chung Yuan Christian University, Taoyuan City, Taiwan; 5 Department of Family and Community Medicine, Chung Shan Medical University Hospital, Taichung, Taiwan

## Abstract

Flunarizine (fz) causes side effects such as movement disorders (MDs). We investigated risk factors associated with fz-related MDs. Participants were recruited from the longitudinal health insurance databases and included patients who took fz for more than 1 month. Patients with one of the underlying diseases, or with concomitant drug use (antipsychotics, metoclopramide or reserpine), and those diagnosed with MDs before fz use were excluded. Fz-related MD was defined as a new diagnosis of parkinsonism or hyperkinetic syndrome including dyskinesia or secondary dystonia during fz use or within 3 months after drug discontinuation. After exposure, 288 individuals had fz-related MDs (parkinsonism, n = 240; hyperkinesia, n = 48). Risk factors associated with these disorders were higher-dose exposure (cumulative defined daily dose [cDDD] ≥87.75, odds ratio [OR]: 3.80; 95% CI: 2.61–5.52), older age (OR: 1.07; 95% CI: 1.06–1.09), history of essential tremor (OR: 6.39; 95% CI: 2.29–17.78) and cardiovascular disease (CVD) (OR: 1.47; 95% CI: 1.14–1.9). The optimal value of cDDD to predict MDs was 58.5 (sensitivity: 0.67, specificity: 0.60), indicating an overall exposure of 585 mg. Higher exposure dose and duration, older age, history of essential tremor, and CVD were associated with fz-associated MDs. Clinicians ought to watch for extrapyramidal side effects when prescribing fz.

## Introduction

Drug-induced parkinsonism (DIP) is a common cause of secondary parkinsonism. Fz, a derivative from cinnarizine represents one of the most common causes of DIP^1^. It is a calcium channel blocker which is frequently prescribed for vertigo, migraine prophylaxis and cerebrovascular insufficiency. The first case of fz-induced parkinsonism was reported in 1984^2^. From that moment, many related cases have been reported^1,3–8^. Apart from its calcium entry blocking properties, fz also has anti-histaminic, anti-serotoninergic and anti-dopaminergic properties. Because of the D2 receptor blocking activity^9^, fz can cause MDs including parkinsonism, orobuccolingual dyskinesia, dystonia, and akathisia^5,10^.

Previous case series showed that elderly women and history of essential tremor may probably serve as risk factors for the development of MDs^4,5,7,11^. By comparing 24 cases of cinnarizine-induced parkinsonism with referred cases of Parkinson’s disease, Santiago and colleagues concluded that aging and essential tremor were possible risk factors for the development of the drug side effects^4^. In a study comparing antipsychotics, Bezerra found that patients with fz or cinnarizine-induced parkinsonism (n = 47) were older^11^. Martí-Massó and colleagues reported that most cases of cinnarizine-induced parkinsonism (about 89%) were females^5^. However, such conclusions may be challenged because of the small study populations.

Until now, studies about fz-related MDs have made use of small sample sizes. Based on previous literature, we hypothesized that (1) older age, gender and comorbidities including essential tremor would increase susceptibility to fz-related MD. (2) Fz might cause MD in a dose-dependent manner due to its D2 receptor blocking activity. The aim of this study was to investigate risk factors associated with fz-related MDs using the national health insurance database.

## Results

Table 1 shows basic characteristics of study participants. The final analysis included 10,020 individuals. Of the overall participants, there were 288 cases of fz-related MDs and 9,732 uninfected individuals. 240 of the 288 cases developed parkinsonism while 48 had hyperkinesia syndrome. Patients with parkinsonism were older and had higher rates of essential tremor than were those with hyperkinesia and the controls. In addition, they were found to have received the highest dose of fz (mean cDDD = 210.79 ± 298.97) followed by patients with hyperkinesia (mean cDDD = 114.33 ± 204.30) and the controls (mean cDDD = 100.68 ± 213.72). Table 2 shows the risk factors for fz-related MDs using multivariate logistic regression analysis. Higher cDDD, older age (OR, 1.07; 95% CI, 1.06–1.09, *p* < 0.0001), history of essential tremor (OR, 6.39; 95% CI, 2.29–17.78, *p* = 0.0004), and baseline CVD (OR, 1.47; 95% CI, 1.14–1.9, *p* = 0.004) were associated with higher risk of fz-related MDs.

**Table 1:** Basic characteristics and comorbidities of patients who received flunarizine.

	Control	Parkinsonism	hyperkinesia	p-value
N	9732	240	48	
Sex				0.1083
Female	5810(59.70%)	128(53.33%)	31(64.58%)	
Male	3922(40.30%)	112(46.67%)	17(35.42%)	
Age at the first intervention (Mean ± SE)	59.10 ± 9.81	68.82 ± 8.46	59.94 ± 9.32	<0.0001
Low-income				
Yes	53(0.54%)	2(0.83%)	0(0.00%)	0.7320
Urbanization				0.2420
Urban	5912(60.75%)	130(54.17%)	27(56.25%)	
Normal	2834(29.12%)	84(35.00%)	17(35.42%)	
Rural	986(10.13%)	26(10.83%)	4(8.33%)	
Co-mobidities				
CKD	136(1.4%)	4(1.67%)	0(0.00%)	0.6681
Severe liver dysfunction	83(0.85%)	0(0.00%)	1(2.08%)	0.2289
History of essential tremor	30(0.31%)	5(2.08%)	0(0.00%)	<0.0001
Other movement disorders	26(0.27%)	1(0.42%)	1(2.08%)	0.0545
DM	1846(18.97%)	56(23.33%)	9(18.75%)	0.2352
CVD	2018(20.74%)	91(37.92%)	13(27.08%)	<0.0001
Flunarizine cDDD (Mean ± SE)	100.68 ± 213.72	210.79 ± 298.97	114.33 ± 204.30	<0.0001
Flunarizine days (Mean ± SE)	150.31 ± 310.20	303.95 ± 470.31	182.73 ± 301.25	<0.0001
Intensity of Flunarizine, cDDD/day (Mean ± SE)	0.82 ± 1.45	0.90 ± 0.58	0.79 ± 0.55	0.6920

N: number; SE: standard error; CKD: chronic kidney disease; DM: diabetes mellitus; CVD: cardiovascular disease, cDDD: cumulative daily defined dose.

**Table 2:** Multivariate logistic regression analysis for flunarizine-related movement disorders.

	OR	95% C.I.	p-value
Flunarizine (ref: cDDD < 29.25)			
29.25 ≤ cDDD < 58.5	1.62	1.06–2.46	0.03
58.6 ≤ cDDD < 87.75	3.02	1.94–4.69	<0.0001
≥87.75	3.80	2.61–5.52	<0.0001
Sex (ref: Female)			
Male	1.00	0.78–1.27	0.99
Age (per 1 year)	1.07	1.06–1.09	<0.0001
Low-income (ref: No)			
Yes	0.97	0.22–4.27	0.97
Urbanization (ref: Urban)			
Normal	1.20	0.92–1.55	0.18
Rural	0.84	0.56–1.25	0.39
Comorbidity at baseline (ref: Without)			
CKD	0.64	0.23–1.78	0.39
Severe liver dysfunction	0.33	0.05–2.41	0.27
History of essential tremor	6.39	2.29–17.78	0.0004
Other movement disorders	1.96	0.44–8.65	0.38
DM	0.94	0.71–1.26	0.69
CVD	1.47	1.14–1.9	0.004

cDDD: cumulative daily defined dose; SE: standard error; CKD: chronic kidney disease; DM: diabetes mellitus; CVD: cardiovascular disease.

**Figure 1:**
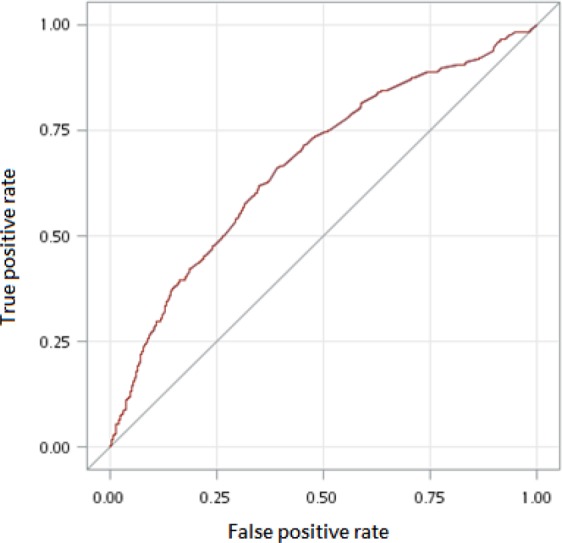
The receiver operating characteristic curve to predict movement disorders according to the cumulative defined daily dose (cDDD) of flunarizine.

Figure 1 displays the ROC curve used to predict MDs based on the cumulative defined daily dose (cDDD) of flunarizine. The area under the curve (AUC) was 0.67 (0.64–0.70) while the optimal predictive value was 58.5 (sensitivity = 0.67, specificity = 0.60). The ROC curve for the exposure duration and intensity showed an optimal predictive value of 98 days (sensitivity = 0.59, specificity = 0.67, AUC = 0.66) and 0.746 cDDD/day (sensitivity = 0.55, specificity = 0.55, AUC = 0.55).

Table 3 shows the risk for MDs according to exposure, dose and duration of fz. In Model 1, higher cDDD of fz was associated with MDs after adjusting for sex, age, low- income, urbanization, and comorbidities. Compared with the reference group (Fz < 29.25 cDDD), the odds ratios were 3.80, 3.02, and 1.62 for cDDD ≥87.75, 58.5 ≤ cDDD < 87.75 and 29.25 ≤ cDDD < 58.5 (*p* < 0.05), respectively. The dose and duration of fz exposure were considered as independent variables in model 2. Longer exposure to fz was associated with increased risk of MDs. The ORs were 4.30, 3.39, 1.90 for exposures ≥147, 98 to <147 and 49 to <98 days, respectively (*p* < 0.05). However, the intensity of fz was not significantly related to the development of MDs. The ORs were 1.87, 1.07, and 0.63 for ≥1.12, 0.75 to <1.12, and 0.37 to <0.75 cDDD/day, *p* > 0.05. Table 4 shows the ORs for MDs stratified by cDDD, DM, and CVD. No obvious interaction was noted.

**Table 3:** Odds ratios for movement disorders associated with flunarizine exposure.

	OR	95% C.I.	p-value
Model 1^¶^			
Flunarizine (ref: cDDD < 29.25)			
29.25 ≤ cDDD < 58.5	1.62	1.06–2.46	0.03
58.6 ≤ cDDD < 87.75	3.02	1.94–4.69	<0.0001
≥87.75	3.80	2.61–5.52	<0.0001
Model 2^¶^			
Flunarizine duration* (ref: <49 days)			
49 to >99 days	1.90	1.31–2.76	0.0007
99 to >147 days	3.39	2.28–5.05	<0.0001
≥147 days	4.30	3.09–5.99	<0.0001
Intensity of Flunarizine^§^ (ref: <0.373 mg/day)			
0.373 to >0.746 mg/day	0.63	0.43–0.91	0.01
0.747 to >1.119 mg/day	1.07	0.74–1.56	0.71
≥1.119 mg/day	1.87	1.25–2.80	0.002

^¶^Model 1 and Model 2 were adjusted for the same covariates, which included sex, age, low- income, urbanization, and comorbidities (chronic kidney disease, severe liver dysfunction, history of essential tremor, other movement disorders, diabetes mellitus, and cardiovascular diseases).

*Total exposure days of flunarizine.

^§^Intensity means the average daily prescribed dose, expressed as cDDD divided by duration.

**Table 4:** Odds ratio for flunarizine-related movement disorders based on DM and CVD.

	Without DM			With DM		
	OR	95% C.I.	p-value	OR	95% C.I.	p-value
Flunarizine (ref: cDDD < 29.25)						
29.25 ≤ cDDD < 58.5	1.84	1.13–3.00	0.01	1.05	0.44–2.53	0.90
58.5 ≤ cDDD < 87.75	3.27	1.96–5.48	<0.0001	2.40	1.01–5.70	0.05
≥87.75	4.20	2.70–6.55	<0.0001	2.92	1.45–5.92	0.003
Interaction p	0.73					
	**Without CVD**			**With CVD**		
Flunarizine (ref: cDDD < 29.25)						
29.25 ≤ cDDD < 58.5	2.05	1.18–3.55	0.0100	1.09	0.55–2.16	0.80
58.5 ≤ cDDD < 87.75	3.89	2.19–6.91	<0.0001	2.01	0.99–4.10	0.05
≥87.75	4.98	3.01–8.23	<0.0001	2.60	1.47–4.58	0.001
Interaction p	0.39					

^¶^Adjusted for sex, age, low-income, urbanization, and comorbidities (chronic kidney disease, severe liver dysfunction, history of essential tremor, other movement disorders, diabetes mellitus, and cardiovascular diseases). DM: diabetes mellitus; CVD: cardiovascular disease; cDDD: cumulative daily defined dose.

## Discussion

To our knowledge, this is the first nationwide study to identify risk factors associated with fz-related MDs. 83.3% of patients with MDs were those with parkinsonism. We found that higher total exposure dose, older age, essential tremor, and CVD were risk factors for MDs following exposure to fz. In this study, the duration of fz exposure was more important than the average daily dose.

In the current study, fz exposure showed a dose-dependent association with MDs. The OR for MDs were 3.80, 3.02 and 1.62 for ≥87.75 cDDD, 58.5 to <87.75 cDDD, and 29.25 to <58.5 cDDD, respectively (*p* < 0.05). MD risk was higher for longer exposure compared with the average daily exposure dose. Fz 58.5 cDDD was the optimal cutoff value (indicating 585 mg, sensitivity = 0.67, specificity = 0.60) while the duration of fz was 98 days (sensitivity = 0.59, specificity = 0.67). Previous case series have found positive associations between duration of fz or cinnarizine use and the development of parkinsonism^5,10,11^. The average onset of MDs ranges from 6–48 months following exposure to fz or cinnarizine^4,9,10^. Fz or cinnarizine use for more than 6 months can block more D2 receptors and ultimately lead to MDs^9^. In addition to the D2 receptor blocking agent, loss of tyrosine hydroxylase in monoaminergic presynaptic neuron may lead to dopamine deletion and can cause MDs^4,5,7^. Our study has not only demonstrated a dose-response effect but has also shown that duration of exposure is a key element to consider when assessing the risk of MDs. Unlike the duration of fz, its intensity was not strongly associated with MD. This may be due to the relatively narrow ranges of the daily prescription doses in clinical practice (usually 5 or 10 mg daily).

Increased risk of fz-related MDs was associated with essential tremor (OR, 6.39; 95% CI, 2.29–17.78, *p* = 0.0004). Essential tremor had a stronger association with fz-related parkinsonism than hyperkinesia. Based on previous literature, a family history of essential tremor or Parkinson’s disease might be a risk factor for fz or cinnarizine-induced parkinsonism^4,12^. About 12.5–38.5% of patients with fz or cinnarizine-induced parkinsonism had a history of essential tremor^4,7^. Both authors speculated that essential tremor results from neurotoxicity of fz on a background inherited predisposition^4,12^. Because idiosyncratic vulnerability has been observed in clinical practice, genetic factors are also thought to be involved in the development of drug-induced parkinsonism^3,6,13,14^. In this study, the outcome included Parkinson’s disease (identified using the ICD-9-CM code), a known condition that is linked with essential tremor^15^. Essential tremor has been associated with increased risk of idiopathic Parkinson’s disease (IPD) although the pathophysiology is not fully understood^15^. We included ICD-9-CM code of Parkinson’s disease for two reasons. First, it is hard to distinguish between fz-induced parkinsonism and idiopathic Parkinson’s disease (IPD) based solely on clinical criteria^3–7^. Miguel and colleagues reported that 43% (13/30) of patients with fz-induced parkinsonism had a clinical pattern similar to IPD patients^16^. The dopaminergic treatment was effective. Clinicians may misclassify fz-induced parkinsonism as IPD. Second, several lines of evidence showed that patients with fz or cinnarizine-induced parkinsonism were not fully recovered after stopping medications while others ultimately developed IPD^5,7^. Based on our data, essential tremor may be related to fz-induced parkinsonism. More investigations are needed to clarify these findings.

Age was another significant risk factor for the fz-induced MDs (OR, 1.073 per 1-year increase in age; 95% CI, 1.06–1.086, *p* < 0.0001). Fz-related MDs have been reported mainly in elderly patients^4,9,11^. Side effects should be considered when prescribing fz to elderly patients. Most studies have shown that fz or cinnarizine-induced MDs occur mainly in women^5,10,11^. Such findings do not align with those presented in this study. The differences may be due to ethnic variation as observed in patients with IPD^17^ or neuroleptic-induced MD^18^.

We also found an association between CVD and fz-related MDs. Atherosclerosis and IPD share a common pathophysiology^19^. CVD is also associated with cerebral small-vessel disease, a known factor that increases the risk of parkinsonism^20^. In this study, there was no interaction between CVD and cumulative dose of fz (Table 4). More investigations would help to clarify this association, as well as the possible mechanism.

In contrast to previous studies, the current study takes advantage of a large dataset, being able to identify over 200 cases of flunarize-induced motor side effects. Besides the sample size, another study strength includes a careful definition of exposure and outcomes. Some of the previous studies have focused mainly on the demographics of fz or cinnarizine-related MDs without including a control group^5,7^. Other studies included cases with IPD or antipsychotic-induced parkinsonism^4,11^. All of these studies have made use of small sample sizes. This study had some limitations. First, the NHIRD does not contain clinical data. Some of the patients may have been wrongly classified. For example, patients who had other secondary parkinsonism syndromes such as progressive supranuclear palsy, or those that had clinical presentations before the index date but never sought medical advice. However, most of the diseases or drugs that may cause parkinsonism, dyskinesia or dystonia were excluded from this study. Although no clinical information was available in the database, our study design probably helped to minimize possible biases. Second, some authors have considered clinical improvement after drug withdrawal as one of the diagnostic criteria of DIP^5^. This information was not also available in the NHIRD. However, some researchers found that patients would not fully recover after being exposed to fz and some patients ultimately have been diagnosed with IPD^5,7,21^. Therefore, it is wise not to consider clinical improvement as one of the criteria for diagnosis of fz-related MDs.

In conclusion, fz-related MDs are associated with a high-dose exposure, longer exposure duration, older age, history of essential tremor, and CVD. Fz is frequently prescribed for vertigo, migraine prophylaxis and cerebrovascular blood flow insufficiency. When considering long-term use (e.g. more than 3 months), physicians should weigh the efficacy and adverse effects of the drug.

## Materials and Methods

### Data Source

This nested case-control study used data from the 2005 and 2010 Longitudinal Health Insurance Databases (LHIDs). Each database contains the original claim data of 1,000,000 beneficiaries randomly sampled from the 23.68 million individuals registered in the NHIRD. 2005 and 2010 represent the year of enrollment of the beneficiaries. The NHIRD contains a comprehensive health care information including diagnoses, prescriptions, and information on inpatient and outpatient care. It covers over 99% of the total population from 1996–2011. The Institutional Review Board of Chung-Shan Medical University Hospital approved this study. All methods were performed in accordance with the relevant guidelines and regulations.

### Definition of exposures

Patients included 55,717 individuals who took fz from 2002–2011 for more than 1 month. The first prescription day was defined as the index date. Excluded from the study were patients aged 45 or younger (n = 28,692) or those who were diagnosed with one of the following diseases before the index date. They included: dementia (international classification of diseases, 9th revision, clinical modification (ICD-9-CM) 290.0~290.43), neurodegeneration (ICD-9-CM 333.0, 333.4, 334.0–334.9), hydrocephalus (ICD-9-CM 331), subdural hemorrhage (ICD-9-CM 432.1), brain tumor (ICD-9-CM 191), Wilson’s disease (ICD-9-CM 275.1), hypoparathyroidism (ICD-9-CM 252.1, 252.8, 252.9, 275.49 A), pantothenate kinase-associated neurodegeneration (ICD-9-CM 277.9I), human immunodeficiency virus infection (ICD-9-CM 042, 079.53, 795.71), neurosyphilis (ICD-9-CM 094.89, 094.9), progressive multifocal leukoencephalopathy (ICD-9-CM 046.3), toxoplasmosis (ICD-9-CM 130.0, 130.7), stroke (ICD-9-CM 431, 432.9, 434, 436), parkinsonism, dyskinesia and dystonia (ICD-9-CM 332.0, 332.1, 333.90, 333.99, 333.7 and 333.8). To minimize drug interaction, patients who took both fz and cinnarizine (n = 892) or in combination with one of the antipsychotic agents (anatomical therapeutic chemical code N05A), metoclopramide or reserpine; (n = 16,113) were excluded.

To determine the dose-response effect, the defined daily dose (DDD) of fz (10 mg per day, determined by the World Health Organization collaborating center) was used. The cumulative DDD (cDDD) was calculated as the total exposure dose divided by the DDD. Duration was defined as the total number of days for the prescription. Drug intensity was determined as cDDD divided by duration, indicating the average daily prescribed dose.

### Definition of Outcomes

Fz-related MDs were defined as a diagnosis of parkinsonism (ICD-9-CM 332.0, 332.1), or hyperkinesia including dyskinesia or symptomatic dystonia (ICD-9-CM 333.90, 333.99, 333.7 and 333.8) from the index date to 3 months after discontinuation of fz. All patients were followed until December 2011, death, or 3 months after discontinuation of fz.

Possible confounders included sex, age, low-income, urbanization, and co-morbidities (Diabetes mellitus (DM, ICD-9-CM: 250), chronic kidney disease (CKD, ICD-9-CM: 585), severe liver dysfunction (ICD-9-CM: 572.2, 571.5, 572.2–572.4), history of essential tremor (ICD-9-CM: 333.1), history of other movement disorders (ICD-9-CM: 333.2, 333.3, 333.5, 333.6), and CVD (ICD-9-CM: 410–414, 433, 444).

### Statistical Analysis

Data were analyzed using the SAS software. Student’s t-test was used to compare the mean difference among groups while chi-square test was used for the nominal variables. The ORs and 95% confidence interval (CI) were estimated using logistic regression models. *P* value < 0.05 was considered statistically significant. The receiver operating characteristic (ROC) curve was used to predict MDs based on cDDD, duration and intensity of fz. These variables were further divided into four categories. The interval for each group was equal to half of the optimal predictive value.

## Data Availability

The data source is included within the manuscript.
